# Simultaneous determination of hydroquinone, catechol, and resorcinol with an electrochemical sensor based on poly-l-valine, multi-walled carbon nanotubes, and Co_3_O_4_ nanoparticles

**DOI:** 10.1007/s00216-025-05923-y

**Published:** 2025-05-26

**Authors:** Noor Raad Abdulrasool, İrem Okman Koçoğlu

**Affiliations:** Department of Chemistry, Faculty of Science, Karabük University, 78050 Karabük, Turkey

**Keywords:** Electrochemical sensor, Dihydroxybenzene isomers, Carboxylated multi-walled carbon nanotubes, Co_3_O_4_ nanoparticles, Poly-l-valine

## Abstract

An electrochemical sensor based on carboxylated multi-walled carbon nanotubes (c-MWCNT), Co_3_O_4_ nanoparticles (Co_3_O_4_NP), and poly-l-valine (PolyVal) was fabricated for the simultaneous determination of hydroquinone (Hyd), catechol (Cat), and resorcinol (Res). The optimum amounts of modification materials on the electrode surface and the optimum pH of the working solution were determined to achieve the best electrode performance. Under optimum conditions, individual determination of each analyte was performed in the presence of other analytes. Simultaneous determination of Hyd, Cat, and Res was also carried out, and the performance characteristics of the electrode, such as working range, LOD, reusability, reproducibility, and selectivity, were evaluated for each analyte. In the simultaneous determination for Hyd, Cat, and Res, the linear working ranges were 5–260 µM, 5–280 µM, and 31–550 µM, and the LOD values were 3.62 µM, 2.57 µM, and 7.97 µM, respectively. The presented electrochemical sensor was successfully applied to the simultaneous determination of Hyd, Cat, and Res in tap water and river water, and satisfactory recovery values were obtained.

## Introduction

Phenolic compounds are widely found in nature as they are frequently used in industrial and agricultural fields [1]. Dihydroxybenzene isomers hydroquinone (Hyd, 1,4-dihydroxybenzene), catechol (Cat, 1,2-dihydroxybenzene), and resorcinol (Res, 1,3-dihydroxybenzene) are intermediates commonly encountered in the production of dyes, polymers, cosmetics, pesticides, photography chemicals, and pharmaceuticals [2–4]. Due to their widespread use and low degradability, they can coexist in natural water and wastewater [4–6]. Since these compounds are highly toxic to humans and harmful to the environment, they are classified as priority toxic pollutants by the United States Environmental Protection Agency and the European Union [3, 5, 7]. Excess intake of Hyd, Cat, and Res may cause headaches, fatigue, tachycardia, cancer, central nervous system problems, liver damage, or kidney damage [8–10]. Therefore, a fast, easy, and reliable method for the determination of these compounds is critical [6, 11].

Various analytical methods, including spectrophotometry [12], fluorescence [13], high-performance liquid chromatography [14], gas chromatography/mass spectrometry [15], and chemiluminescence [16], have been employed for the determination of these compounds. Although these methods are reliable, they are time-consuming, expensive, and complex [3, 11]. Electrochemical sensors offer a simpler, faster, and more sensitive alternative [17, 18]. Hyd, Cat, and Res are electroactive species due to the hydroxyl groups present in their structure and can be detected by electrochemical sensors [19, 20]. However, their structural similarities lead to overlapping redox peaks, making simultaneous detection on bare electrodes difficult [9, 10, 19]. Therefore, electrode modification is of great importance for the selective and sensitive simultaneous determination of these compounds [4, 9, 20]. For the simultaneous determination of Hyd, Cat, and Res, various modification materials, such as carbon nanomaterials [7, 17], metal nanoparticles [10, 19], metal oxide nanoparticles [18, 21], and polymeric films [5, 11] have been used for electrode modification.

The use of carbon nanomaterials as electrode modification materials in electrochemical sensors is widespread due to their excellent conductivity, chemical stability, and biocompatibility [22, 23]. Among these materials, carbon nanotubes (CNTs), particularly multi-walled carbon nanotubes (MWCNTs), offer a large surface area, a wide electrochemical window, fast electron transfer, and mechanical strength [24–26]. These properties provide improved performance characteristics of electrochemical sensors, including sensitivity, limit of detection, and stability [25]. Carboxylated multi-walled carbon nanotubes (c-MWCNTs), which are obtained by functionalizing MWCNTs with a carboxyl group (− COOH), further improve dispersion and biocompatibility in composite systems [27, 28].

Metal oxide nanomaterials are also attracting interest for sensor applications due to their high surface area/volume ratio, stability, biocompatibility, and superior electrocatalytic activity [29, 30]. Co_3_O_4_ nanoparticles (Co_3_O_4_NPs) are effective modification materials for their good reversible redox capability and low cost [31]. The coexistence of Co(II) and Co(III) in the Co_3_O_4_ nanostructure is considered to increase the electron transfer process on the electrode surface, as it provides the diversity of polar regions [29, 32].

Amino acid–based polymers provide functional groups (–COOH, –NH_2_, –OH) that promote interaction with analytes and improve electrochemical performance [33, 34]. Poly-l-valine (PolyVal) obtained by polymerization of l-valine, a branched-chain amino acid, exhibits strong catalytic activity towards various analytes as a result of the reactive groups present in its structure [35, 36]. Among various methods for the polymerization of amino acids, electropolymerization stands out as a simple and effective method [33, 37].

In this study, a novel electrochemical sensor for the simultaneous determination of Hyd, Cat, and Res was developed by modifying the glassy carbon electrode (GCE) surface with c-MWCNT, Co_3_O_4_NP, and PolyVal. The combination of these three materials has not been reported before for the simultaneous detection of dihydroxybenzene isomers. The c-MWCNT − Co_3_O_4_NP/PolyVal/GCE was prepared by electropolymerization of l-valine on the GCE surface, followed by drop-coating the dispersion of c-MWCNT and Co_3_O_4_NP in chitosan solution on the surface. The amounts of modification materials on the electrode surface and the pH of the working buffer were optimized to achieve the best analytical performance of the electrochemical sensor. Under optimum conditions, individual determination of each analyte in the presence of other analytes, as well as simultaneous determination of the analytes, was performed. Performance characteristics such as LOD, working range, reusability, reproducibility, and selectivity were determined. To test the analytical applicability of the electrochemical sensor, simultaneous determination of Hyd, Cat, and Res in tap water and river water was carried out, and remarkable results were obtained.

## Experimental

### Reagents

Catechol, sodium dihydrogen phosphate, disodium monohydrogen phosphate, chitosan, potassium chloride, sodium chloride, potassium hexacyanoferrate(III), potassium hexacyanoferrate(II) trihydrate, citric acid, ascorbic acid, sodium sulfate, acetic acid, phosphoric acid, and Co_3_O_4_ nanoparticles were obtained from Sigma-Aldrich. Hydroquinone, β-naphthol, boric acid, magnesium chloride hexahydrate, and calcium chloride were purchased from Merck. Resorcinol was received from Acros Organics. l-Valine was bought from Thermo Fisher Scientific, glucose from Fluka, nickel(II) sulfate hexahydrate from Carlo Erba, and c-MWCNT from Cheap Tubes Inc.

### Instrumentation

Electrochemical studies were conducted on a PalmSens EmStat^3^ electrochemical analyzer (PalmSens BV, the Netherlands). A three-electrode system consisting of c-MWCNT − Co_3_O_4_NP/PolyVal modified glassy carbon electrode (GCE) (3.0 mm diameter, ItalSens, the Netherlands) as the working, Ag/AgCl (ItalSens, the Netherlands) as the reference, and Pt wire (ItalSens, the Netherlands) as the auxiliary electrode was used for the electrochemical measurements. Cyclic voltammetry (CV) studies were carried out in 5 mM [Fe(CN)_6_]^3−/4−^ solution containing 0.1 M KCl in the potential range of − 0.40 to + 0.80 V at a 50 mVs^−1^ scan rate. Differential pulse voltammetry (DPV) measurements were performed in 0.025 M Britton-Robinson buffer solution (BRBS) at pH 5.0 with the following parameters: pulse potential 50 mV, pulse time 50 ms, step potential 4 mV, and scan rate 40 mV s^−1^. Scanning electron microscopy (SEM) studies were conducted with a FEI Quanta 450 FEG model scanning electron microscope (acceleration voltage: 10.00 kV).

### Preparation of the c-MWCNT − Co_3_O_4_NP/PolyVal/GCE

**Scheme 1:**
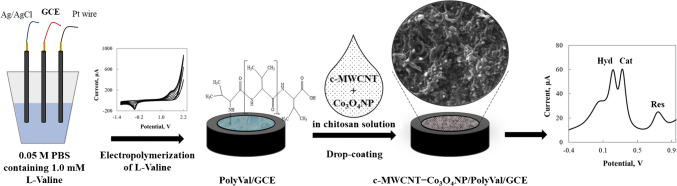
Preparation steps of the c-MWCNT − Co_3_O_4_NP/PolyVal/GCE

For electropolymerization of l-valine on a clean GCE surface, 1.0 mM l-valine solution was prepared in 0.05 M PBS with a pH of 6.5. Cyclic voltammograms were recorded in this solution in the potential range of − 1.2 to + 2.2 V with a scan rate of 100 mV s^−1^ for 10 cycles, and PolyVal/GCE was obtained [38]. The electrodes were then cleaned with distilled water to remove residual monomers. To prepare the dispersion containing c-MWCNT and Co_3_O_4_NP, chitosan solution of 10 mg mL^−1^ prepared in acetate buffer with pH 4.0 was used. Then, 7.5 mg c-MWCNT and 7.5 mg Co_3_O_4_NP were sonicated in 1.0 mL chitosan solution for 4 h. After vortexing for 10 min, 5 µL of the c-MWCNT − Co_3_O_4_NP − chitosan mixture was dropped onto the PolyVal/GCE surface and dried at room temperature to obtain c-MWCNT − Co_3_O_4_NP/PolyVal/GCE. A schematic representation of the preparation steps of c-MWCNT − Co_3_O_4_NP/PolyVal/GCE is shown in Scheme 1.

## Results and discussion

### Optimization of the amounts of modification materials on c-MWCNT − Co_3_O_4_NP/PolyVal/GCE

The amount of each modification material on the electrode surface is an important parameter that affects the response of the electrode to the analyte [39]. Therefore, the composition and thickness of the components of the modified layer were optimized. To determine the optimum amount of PolyVal on the surface, the number of cycles during electropolymerization was examined; to determine the optimum amounts of c-MWCNT and Co_3_O_4_NP, their concentrations in the c-MWCNT − Co_3_O_4_NP − chitosan mixture were examined. The amount of each material in the modifier layer was optimized by varying the amount of a single material while keeping the amounts of the other materials constant. In each optimization study, the responses of the electrodes to 0.025 M BRB solutions containing 200 µM each of Hyd, Cat, and Res were examined.

**Fig. 1:**
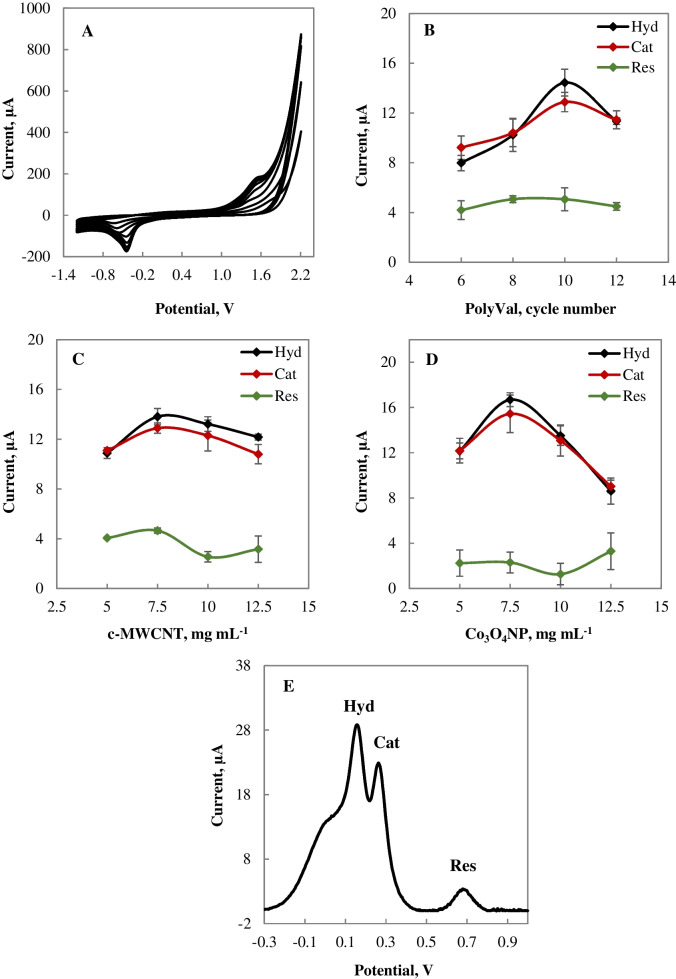
**A** Cyclic voltammograms for the formation of PolyVal on the GCE surface in 0.05 M PBS (pH 6.5) containing 1.0 mM l-valine at a scan rate of 100 mV s^−1^. Effects of **B** cycle number for the electropolymerization of l-valine, **C** c-MWCNT amount, and **D** Co_3_O_4_NP amount on the c-MWCNT − Co_3_O_4_NP/PolyVal/GCE response to 200 µM each of Hyd, Cat, and Res (in 0.025 M BRBS with pH 5.0, error bars represent the standard deviation for each assay, *N* = 3). **E** DPV response of c-MWCNT − Co_3_O_4_NP/PolyVal/GCE prepared with optimum amounts of the modification materials in 0.025 M BRBS (pH 5.0) containing 200 µM each of Hyd, Cat, and Res

**Scheme 2:**
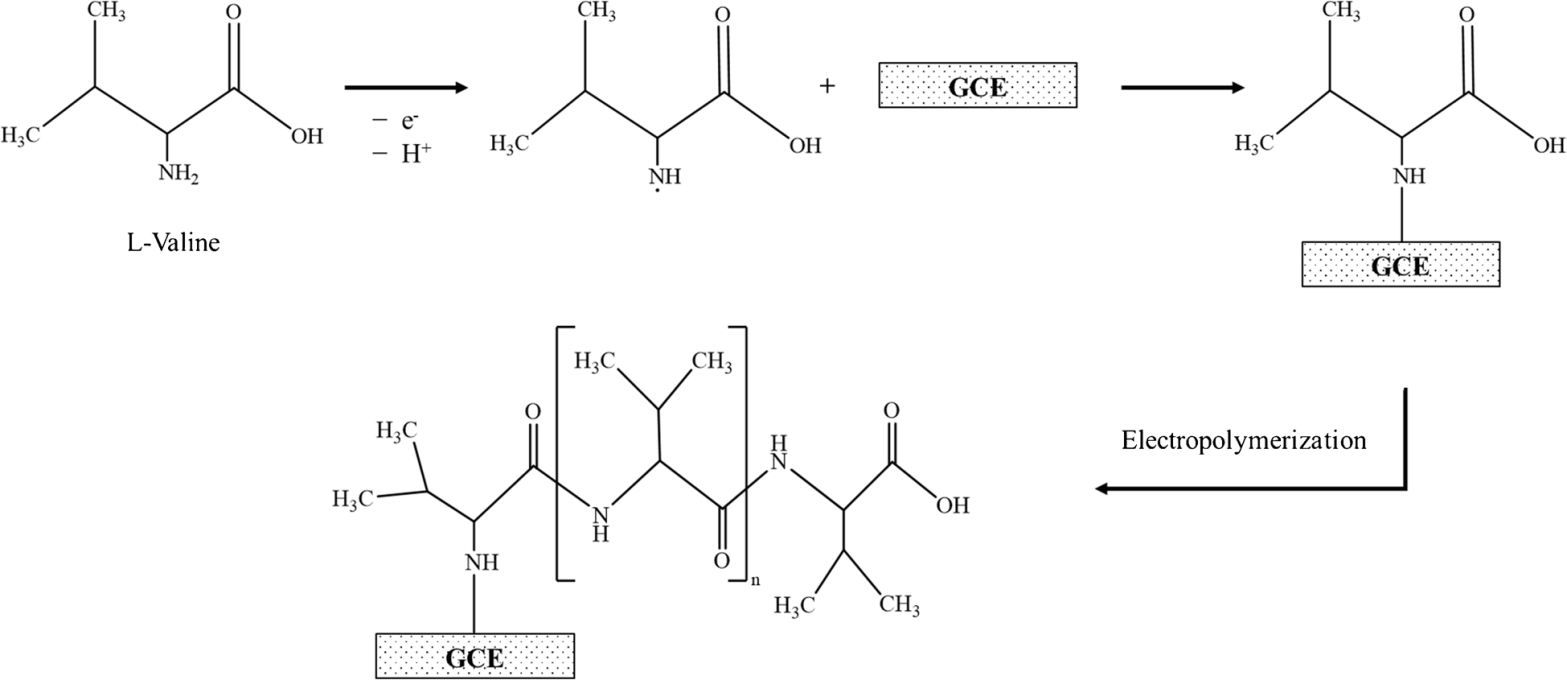
Possible mechanism for electropolymerization of l-valine on GCE surface

The electropolymerization of l-valine on the GCE surface was carried out by cyclic voltammetry with 10 consecutive cycles at a scan rate of 100 mV s^−1^ over a potential range of − 1.2 to + 2.2 V in 0.05 M PBS (pH 6.5) containing 1.0 mM l-valine. Voltammograms recorded for the electropolymerization of l-valine are shown in Fig. 1A. First, at around 1.6 V potential, the amino group in the l-valine monomer is thought to be oxidized to the α-amino radical, which binds to the carbon electrode surface via a carbon–nitrogen bond, and then a polymeric film is formed on the electrode surface in subsequent voltammetric cycles [27, 38, 40]. A possible mechanism for the formation of PolyVal on the GCE surface is presented in Scheme 2 [36, 38, 41]. Since the thickness of PolyVal on the electrode surface is controlled by the number of cyclic voltammograms during the electropolymerization of l-valine [41, 42], the number of cycles in the formation of PolyVal was optimized to increase the response to analytes. Four different modified electrodes were prepared in which PolyVal was formed with 6, 8, 10, and 12 cycles. The responses of the modified electrodes in 0.025 M BRBS containing 200 µM each of Hyd, Cat, and Res were analyzed by DPV, and the peak currents versus the number of cycles were plotted (Fig. 1B). For Hyd and Cat in particular, there was a noticeable increase in peak heights when the number of cycles was increased from 6 to 10, followed by a decrease after 10 cycles. In the Res response, there was no significant difference between the peak currents at 8 and 10 cycles, but there was a decrease at lower and higher cycle numbers. After 10 cycles, the decrease in peak heights was attributed to the prevention of electron transfer by increasing the thickness of the PolyVal film on the electrode surface [37]. Therefore, the optimum cycle number for the electropolymerization of l-valine was chosen as 10.

In order to determine the effect of the amount of c-MWCNT on the Hyd, Cat, and Res responses of the electrochemical sensor, four different mixtures were prepared by varying the concentration of c-MWCNT as 5.0, 7.5, 10.0, and 12.5 mg mL^−1^ in the c-MWCNT − Co_3_O_4_NP − chitosan mixture. The responses of the modified electrodes prepared using these mixtures were analyzed by DPV in 0.025 M BRBS with each analyte at a concentration of 200 µM. The plot of peak currents versus c-MWCNT concentration (Fig. 1C) showed that the peak currents of each analyte increased when the c-MWCNT concentration was increased from 5.0 to 7.5 mg mL^−1^ and decreased at concentrations higher than 7.5 mg mL^−1^, and this amount was chosen as optimum. Similarly, to determine the optimum amount of Co_3_O_4_NP, the amount of Co_3_O_4_NP in 1 mL of c-MWCNT − Co_3_O_4_NP − chitosan mixture was varied to be 5.0, 7.5, 10.0, and 12.5 mg. The responses of the modified electrodes prepared by using each mixture to Hyd, Cat, and Res were plotted against Co_3_O_4_NP concentration (Fig. 1D). The best response to each analyte was obtained with the modified electrode prepared with the mixture at a concentration of 7.5 mg mL^−1^ Co_3_O_4_NP, and this amount was selected as optimum. When the concentration of both c-MWCNT and Co_3_O_4_NP in the c-MWCNT − Co_3_O_4_NP − chitosan mixture was increased above 7.5 mg mL^−1^, the decrease in the responses of the modified electrodes to the analytes was thought to be due to the diffusion limitation problem that occurs as the surface gets thicker [27, 43, 44]. DPV response of c-MWCNT − Co_3_O_4_NP/PolyVal/GCE prepared with optimum amounts of the modification materials in 0.025 M BRBS at pH 5.0 in the presence of 200 µM Hyd, Cat, and Res is presented in Fig. 1E to illustrate the well-separated oxidation peaks of the analytes.

### Optimization of pH

**Fig. 2:**
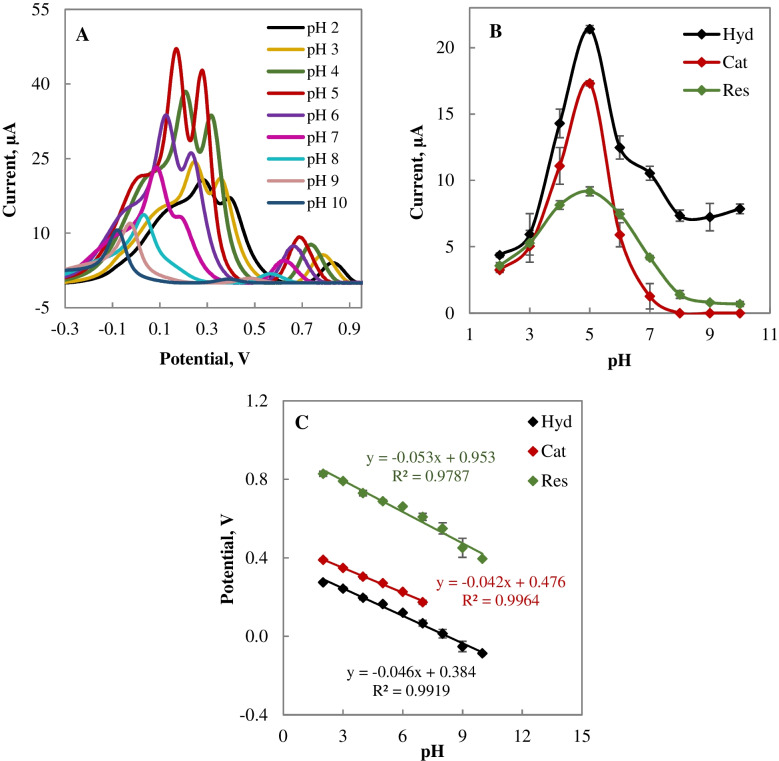
**A** Effect of pH on the c-MWCNT − Co_3_O_4_NP/PolyVal/GCE response to 250 µM each of Hyd, Cat, and Res; **B** peak currents vs. pH plot; and **C** peak potentials vs. pH plot (in 0.025 M BRBS, error bars represent the standard deviation for each assay, *N* = 3)

The pH of the buffer solution has a significant effect on the analytical performance of the modified electrode [45]. To determine the optimum pH of the working solution, nine buffer solutions were prepared by varying the pH of 0.025 M BRBS from 2.0 to 10.0. In each buffer solution, the voltammetric responses of the c-MWCNT − Co_3_O_4_NP/PolyVal/GCE to 250 µM Hyd, Cat, and Res were recorded by DPV (Fig. 2A). The peak currents of the analytes in each buffer solution were plotted against pH and are given in Fig. 2B. The highest response of each analyte and better separation of peaks was obtained in the buffer solution with a pH of 5.0, and this value was chosen as the optimum pH. In addition, the oxidation potentials of the analytes in buffer solutions at each pH were examined. As seen in Fig. 2A, the peak potentials shifted negatively with increasing pH. The plot of peak potentials versus pH for each analyte was observed to be linear (Fig. 2C) and the slopes were 0.046, 0.042, and 0.053 V pH^−1^ for Hyd, Cat, and Res, respectively. The slope values close to the Nernstian theoretical value of 0.059 V pH^−1^ indicate that equal numbers of electrons and protons are involved in the electrode reactions [1, 4, 10].

### Surface morphology

**Fig. 3:**
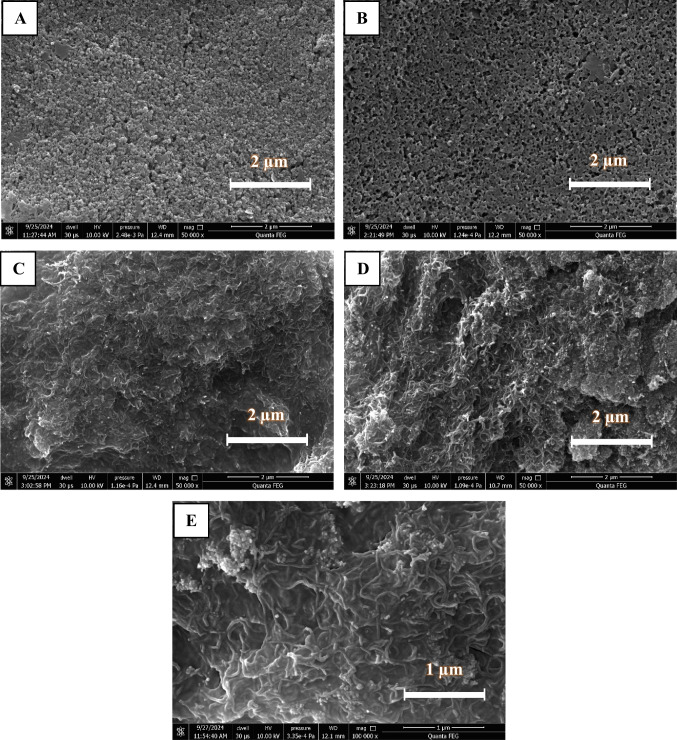
SEM images of **A** SPE, **B** PolyVal/SPE, **C** c-MWCNT/PolyVal/SPE, **D** c-MWCNT − Co_3_O_4_NP/PolyVal/SPE (magnified 50,000 times), and **E** c-MWCNT − Co_3_O_4_NP/PolyVal/SPE (magnified 100,000 times)

Scanning electron microscopy (SEM) technique was used to image the effect of each modification material on the carbon electrode surface. Screen-printed carbon electrodes (SPE) were used as carbon electrodes. SEM images of (A) bare SPE, (B) PolyVal/SPE, (C) c-MWCNT/PolyVal/SPE, (D) c-MWCNT − Co_3_O_4_NP/PolyVal/SPE (magnified 50,000 times), and (E) c-MWCNT − Co_3_O_4_NP/PolyVal/SPE (magnified 100,000 times) are provided in Fig. 3. The surface in image B was obtained by electropolymerization of l-valine on the bare carbon surface shown in image A. It was observed that the PolyVal film was homogeneously coated on the carbon surface, resulting in a smoother surface. The surface after modification of c-MWCNT is displayed in image C. The unique filamentous structure of c-MWCNTs is clearly seen to be homogeneously distributed on the electrode surface. In image D, the spherical structure of Co_3_O_4_NPs and their dispersion between c-MWCNTs can be seen distinctly. Image E shows the c-MWCNT − Co_3_O_4_NP/PolyVal composite on the carbon surface with a higher magnification. The c-MWCNT and Co_3_O_4_NP structures can be seen more prominently in this image. SEM images indicate that each modification step was performed successfully.

### Electrochemical characterization

#### Electrochemical behavior of modified electrodes

**Fig. 4:**
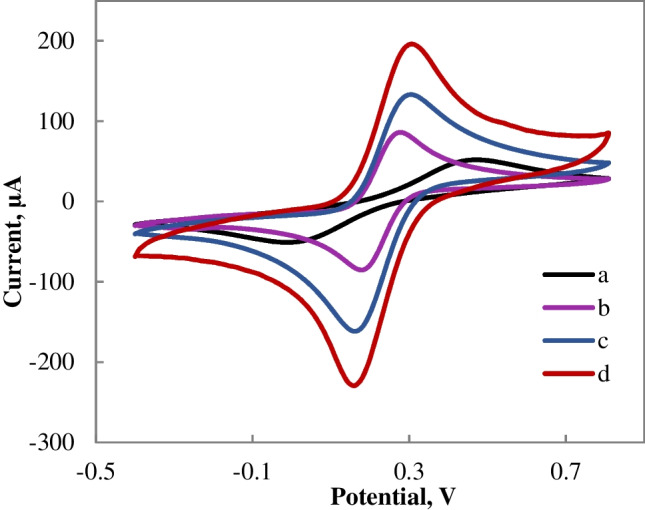
Cyclic voltammograms of (**a**) GCE, (**b**) PolyVal/GCE, (**c**) Co_3_O_4_NP/PolyVal/GCE, and (**d**) c-MWCNT − Co_3_O_4_NP/PolyVal/GCE in a solution of 0.1 M KCl containing 5 mM of Fe(CN)_6_^3−/4^ (scan rate, 50 mVs.^−1^)

The CV method was applied to investigate the electrochemical properties of the modified electrodes in 0.1 M KCl containing 5 mM of Fe(CN)_6_^3−/4^ at a scan rate of 50 mVs^−1^. Figure 4 presents the cyclic voltammograms of (a) GCE, (b) PolyVal/GCE, (c) Co_3_O_4_NP/PolyVal/GCE, and (d) c-MWCNT − Co_3_O_4_NP/PolyVal/GCE. It was observed that the currents of the redox peaks increased at each step of the electrode modification. This may be explained by the fact that the modification materials increase the electron transfer efficiency at the electrode surface. The highest peak current was obtained with c-MWCNT − Co_3_O_4_NP/PolyVal/GCE, which was considered to be due to the synergistic effect that occurs when all modification materials are present together, resulting in improved electrode properties such as electron transfer kinetics, surface area, and electrocatalytic activity [10, 46].

The electroactive surface areas of the electrodes at each modification step were calculated using the Randles–Sevcik equation [47]:

$${I}_{p}=2.69\times {10}^{5}{AD}^{1/2}{n}^{3/2}{\nu }^{1/2}C$$

In the equation, *I*_p_ is the peak current (A), *D* is the diffusion coefficient of Fe(CN)_6_^3−/4−^ (7.6 × 10^−6^ cm^2^ s^−1^), *n* is the number of electrons participating in the redox reaction (1), *ν* is the scan rate (0.05 V s^−1^), *C* is the concentration of Fe(CN)_6_^3−/4−^ in the redox solution (5 × 10^−6^ mol cm^−3^), and *A* is the electrode area (cm^2^). The electroactive surface areas were calculated as 0.062 cm^2^, 0.102 cm^2^, 0.160 cm^2^, and 0.234 cm^2^ for GCE, PolyVal/GCE, Co_3_O_4_NP/PolyVal/GCE, and c-MWCNT − Co_3_O_4_NP/PolyVal/GCE, respectively. These results indicate that the surface areas of the modified electrodes increased at each step owing to the outstanding features of the modification materials.

#### Scan rate effect

**Fig. 5:**
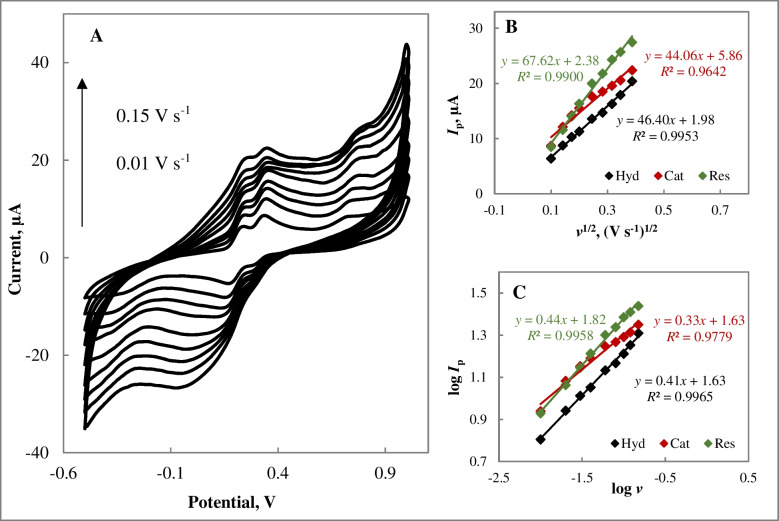
**A** Cyclic voltammograms of c-MWCNT − Co_3_O_4_NP/PolyVal/GCE in 0.025 M pH 5.0 BRBS containing 200 µM each of Hyd, Cat, and Res at various scan rates, **B** *I*_p_ versus *ν*^1/2^ plot, and **C** log *I*_p_ versus log *ν* plot

To determine the electron transfer process between the analytes and c-MWCNT − Co_3_O_4_NP/PolyVal/GCE, the effect of scan rate on the electrochemical behavior of Hyd, Cat, and Res was investigated. For this purpose, cyclic voltammograms were recorded at various scan rates ranging from 0.01 to 0.15 V s^−1^ in 0.025 M BRBS with a pH of 5.0 containing 200 µM each of Hyd, Cat, and Res (Fig. 5A). The oxidation peak currents of Hyd, Cat, and Res increase with increasing scan rate and have a linear relationship with the square root of the scan rate (Fig. 5B), suggesting that the electron transfer mechanism between the analytes and c-MWCNT − Co_3_O_4_NP/PolyVal/GCE is diffusion controlled (1, 4). Furthermore, as seen in Fig. 5C, the logarithms of the anodic peak currents of Hyd, Cat, and Res are linearly proportional to the logarithm of the scan rate with slopes of 0.41, 0.33, and 0.44, respectively. The slopes close to 0.5 also indicate that electron transfers occur through diffusion-controlled processes [48].

### Individual determination of hydroquinone, catechol, and resorcinol

**Fig. 6:**
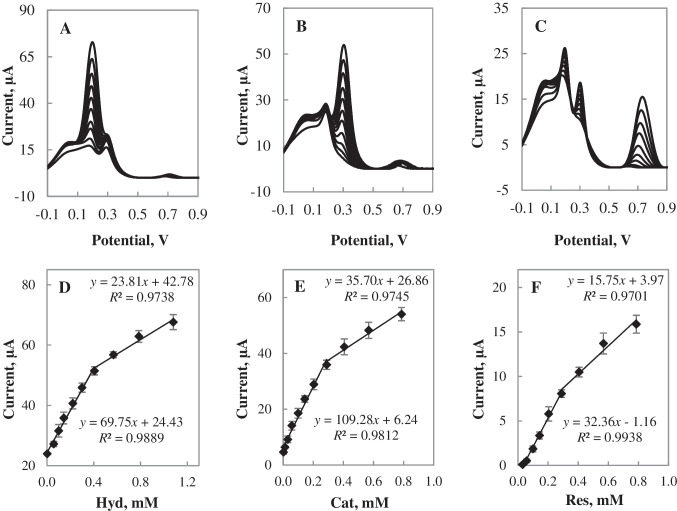
DPV responses of c-MWCNT − Co_3_O_4_NP/PolyVal/GCE in 0.025 M BRBS with a pH of 5.0 **A** to Hyd at different concentrations from 2 to 1100 µM in the presence of 50 µM Cat and 100 µM Res, **B** to Cat at different concentrations from 2 to 800 µM in the presence of 50 µM Hyd and 100 µM Res, and **C** to Res at different concentrations from 30 to 800 µM in the presence of 50 µM Hyd and 50 µM Cat. Plots of peak currents versus **D** Hyd, **E** Cat, and **F** Res concentrations (error bars represent the standard deviation for each assay, *N* = 3)

Individual quantitative determination of each analyte in the presence of other analytes at constant concentration was performed by DPV under optimum conditions. Figure 6A shows the DPV responses of c-MWCNT − Co_3_O_4_NP/PolyVal/GCE to Hyd concentrations from 2 to 1100 µM in 0.025 M BRBS (pH 5.0) containing 50 µM Cat and 100 µM Res. The oxidation peak currents obtained from the voltammogram were plotted against Hyd concentration (Fig. 6D). In the concentration ranges 2 − 400 µM and 400 − 1100 µM, the current responses increased linearly with Hyd concentration. The limit of detection (LOD) for the individual determination of Hyd in the presence of Cat and Res was found to be 0.51 µM. The current responses of c-MWCNT − Co_3_O_4_NP/PolyVal/GCE to Cat in the concentration range of 2 − 800 µM with the existence of 50 µM Hyd and 100 µM Res were analyzed by DPV, and the voltammograms are depicted in Fig. 6B. The peak currents increased with Cat concentration and were found to be linear in two concentration ranges: 2 − 290 µM and 290 − 800 µM, as shown in the calibration plot in Fig. 6E. The LOD for the individual determination of Cat was 0.36 µM. Voltammograms recorded at various concentrations between 30 and 800 µM of Res in solution containing 50 µM Hyd and 50 µM Cat are shown in Fig. 6C. In Fig. 6F, the peak currents were plotted as a function of Res concentration, and two linear ranges were obtained. c-MWCNT − Co_3_O_4_NP/PolyVal/GCE responded linearly to Res in the concentration ranges 30 − 290 µM and 290 − 800 µM with a LOD of 1.94 µM. Two distinct linear ranges were observed for the individual determination of each analyte. The higher sensitivity in the lower concentration range and the decreased slope in the second linear range can be attributed to kinetic limitations occurring at the electrode surface at elevated analyte concentrations [49–51]. On the other hand, voltammograms show that the peak currents of Hyd, Cat, and Res increase with increasing concentrations of these isomers, while there are no significant changes in the peaks of the other analytes. Therefore, it is strongly suggested that Hyd, Cat, or Res can be individually determined in coexistence with other analytes and that other isomers do not affect the determination of each analyte.

### Simultaneous determination of hydroquinone, catechol, and resorcinol

**Fig. 7:**
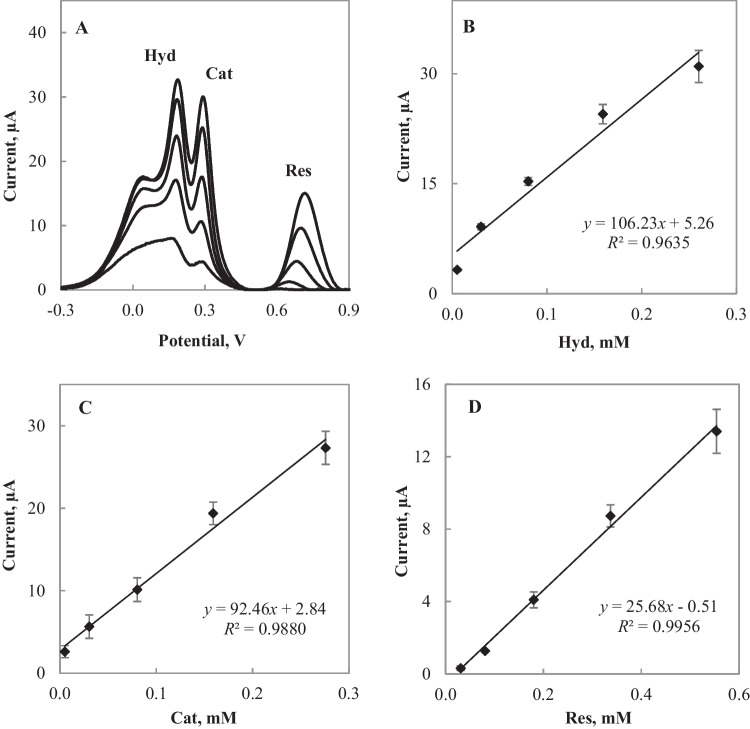
**A** DPV responses of c-MWCNT − Co_3_O_4_NP/PolyVal/GCE to different concentrations of Hyd, Cat, and Res in 0.025 M pH 5.0 BRBS. Plots of peak currents as a function of **B** Hyd concentration, **C** Cat concentration, and **D** Res concentration

Simultaneous determination of Hyd, Cat, and Res was performed by DPV under optimum conditions by simultaneously changing the concentration of each analyte in 0.025 M BRBS with pH 5.0, and voltammograms are given in Fig. 7A. Three well-separated peaks for Hyd, Cat, and Res are clearly seen in the voltammograms. As the concentration of each analyte increases simultaneously, it is apparent that the peak currents also increase. Plots of oxidation peak currents versus Hyd, Cat, and Res concentrations are given in Fig. 7B, C, and D, respectively. The c-MWCNT − Co_3_O_4_NP/PolyVal/GCE responded linearly to Hyd in the concentration range 5–260 µM with a sensitivity of 106.23 µA mM^−1^. The linear working range of Cat was determined as 5–280 µM with a sensitivity of 92.46 µA mM^−1^. The peak currents increased linearly by increasing the concentration of Res in the range 31–550 µM, and the sensitivity was 25.68 µA mM^−1^. LOD values for Hyd, Cat, and Res were 3.62 µM, 2.57 µM, and 7.97 µM, respectively. The results indicated that the simultaneous determination of three dihydroxybenzene isomers, which are difficult to determine simultaneously due to overlapping peaks or interference with the responses of each other, is possible with the presented electrochemical sensor.

To determine the reusability of the c-MWCNT − Co_3_O_4_NP/PolyVal/GCE, five different BRBS (0.025 M, pH 5.0) containing a constant 150 µM of each of Hyd, Cat, and Res were prepared. The DPV responses of c-MWCNT − Co_3_O_4_NP/PolyVal/GCE in each solution were recorded consecutively. The relative standard deviation (RSD) of the five measurements was calculated for each analyte and found to be 2.6%, 2.6%, and 6.7% for Hyd, Cat, and Res, respectively. Five different c-MWCNT − Co_3_O_4_NP/PolyVal/GCE were prepared to establish reproducibility. The current responses of each electrode in 0.025 M pH 5.0 BRBS containing 150 µM each of Hyd, Cat, and Res were analyzed by DPV. RSD values of the peak currents obtained for each analyte with each electrode were calculated. The reproducibility was determined to be 2.4%, 5.8%, and 3.9% for Hyd, Cat, and Res, respectively. Additionally, the storage stability of the c-MWCNT − Co_3_O_4_NP/PolyVal/GCE was evaluated by storing the modified electrode at room temperature for two months. After 1 month, the electrochemical sensor showed a 15.7% decrease in its electrochemical response to Hyd, while no significant changes were observed for Cat and Res. At the end of 2 months, the sensor exhibited a 19.0% and 17.5% decrease in response to Hyd and Cat, respectively, whereas the response towards Res remained largely stable. These results demonstrate that the developed sensor maintains acceptable stability over a practical storage period, especially for Cat and Res.

**Fig. 8:**
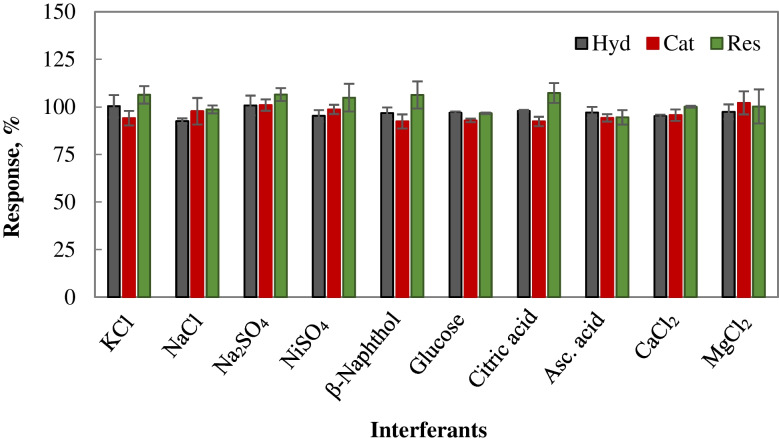
Effect of possible interfering species (100 µM each) on the response of c-MWCNT − Co_3_O_4_NP/PolyVal/GCE to Hyd, Cat, and Res at 100 µM each (in pH 0.025 M BRBS, pH 5.0, *N* = 3)

The effect of some common interfering species (KCl, NaCl, Na_2_SO_4_, NiSO_4_, β-naphthol, glucose, citric acid, ascorbic acid, CaCl_2_, and MgCl_2_) on the response of c-MWCNT − Co_3_O_4_NP/PolyVal/GCE to Hyd, Cat, and Res was analyzed to determine the selectivity of the electrode. The response of the electrode in a solution containing 100 µM each of Hyd, Cat, and Res was compared with the response in the presence of 100 µM of interfering species. As shown in Fig. 8, the effect of each interfering species on the response of the electrode to Hyd, Cat, and Res is less than 10%. These results suggest that the electrochemical sensor has good selectivity towards dihydroxybenzene isomers.

The performance characteristics of several electrochemical sensors reported in the literature for the determination of Hyd, Cat, and Res are given in Table 1. The presented sensor was found to have linear working ranges for the individual determinations of Hyd, Cat, and Res in the presence of other isomers that are wider than most of the studies given in the table and comparable to some of them. In addition, the LOD values were also compatible with the studies in the literature. For the simultaneous determination of Hyd, Cat, and Res, c-MWCNT − Co_3_O_4_NP/PolyVal/GCE was found to have quite wide linear working ranges, while the LOD values were slightly higher than the studies in the literature. In addition to its promising analytical performance, c-MWCNT − Co_3_O_4_NP/PolyVal/GCE is relatively simple to fabricate, involving a drop coating and electropolymerization procedure without the need for complex synthesis steps or expensive reagents, which offers an advantage in terms of reproducibility, low cost, and ease of fabrication compared to some other modified electrodes reported in the literature. Considering all the results, it is concluded that c-MWCNT − Co_3_O_4_NP/PolyVal/GCE can be successfully used for the sensitive and selective determination of Hyd, Cat, and Res, both individually and simultaneously.

**Table 1:** Comparison of the performance characteristics of various electrochemical sensors reported for the individual and simultaneous determination of Hyd, Cat, and Res

Electrode	Analyte	Individual determination		Simultaneous determination		Ref
		Linear range, µM	LOD, µM	Linear range, µM	LOD, µM	
PC_800_/GCE	Hyd	2.8–78.7	0.27	2.8–99.8	0.15	[1]
	Cat	16.9–153.6	0.12	5.7–124.8	0.09	
	Res	–	–	–	–	
KOH-activated GSEC film	Hyd	0.5–250	0.1	0.5–200	0.1	[9]
	Cat	0.5–300	0.1	0.5–200	0.1	
	Res	0.1–500	0.05	0.2–400	0.05	
ERGO-pEBT/AuNPs/GCE	Hyd	0.52–31.4	0.015	3.52–33.6	0.012	[48]
	Cat	1.44–31.2	0.008	3.52–33.6	0.012	
	Res	3.8–72.2	0.039	7.4–49.7	0.022	
TFPB-BD-COF/PtNPs/NH_2_-MWCNT/GCE	Hyd	0.01–1000	–	0.2–360	0.022	[10]
	Cat	0.02–1000	–	0.2–360	0.015	
	Res	2–800	–	4–360	0.26	
Nafion/MWCNTs/CDs/MWCNTs	Hyd	1–200	0.07	20–120	–	[7]
	Cat	4–200	0.06	20–120	–	
	Res	3–400	0.15	20–120	–	
MWCNT@rGONR/GCE	Hyd	5–1160	–	15–921	3.89	[8]
	Cat	5–1165	–	15–1101	1.73	
	Res	10–490	–	15–1301	5.77	
P-rGO/GCE	Hyd	5–90	0.08	–	–	[2]
	Cat	5–120	0.18	–	–	
	Res	5–90	2.62	–	–	
Graphene–chitosan/GCE	Hyd	1–300	0.75	1–400	–	[6]
	Cat	1–400	0.75	1–400	–	
	Res	1–550	0.75	1–400	–	
Au–Pd NF/rGO/GCE	Hyd	1.6–3000	0.50	1.6–100	0.5	[19]
	Cat	2.5–460	0.85	2.5–100	0.8	
	Res	2–290	0.67	2.0–100	0.7	
CoFe_2_Se_4_/PCF-2/GCE	Hyd	0.5–200	0.13	0.5–180	0.53	[17]
	Cat	0.5–190	0.15	1–160	0.84	
	Res	5–350	1.36	10–120	2.62	
ERGO/MWCNTPE	Hyd	3–200		0.4–400	0.028	[52]
	Cat	3–200		0.4–400	0.083	
	Res	–	–	–	–	
Ni/N-GO/GCE	Hyd	1.4–800	0.06	–	–	[53]
	Cat	1–800	0.16	–	–	
	Res	–	–	–	–	
c-MWCNT − Co_3_O_4_NP/PolyVal/GCE	Hyd	2–1100	0.51	5–260	3.62	This work
	Cat	2–800	0.36	5–280	2.57	
	Res	30–800	1.94	31–550	7.97	

*PC*_*800*_, porous carbon prepared at 800℃; *GSEC*, graphene sheets embedded carbon; *ERGO*, electrochemically reduced graphene oxide; *pEBT*, poly(Eriochrome black T); *AuNPs*, gold nanoparticles; *TFPB*, 3,5-tris-(4-formylphenyl) benzene; *BD*, benzidine; *PtNPs*, platinum nanoparticles; *NH*_*2*_*-MWCNT*, amino functionalized multi-walled carbon nanotubes; *CDs*, carbon dots; *rGONR*, reduced graphene oxide nanoribbon; *P-rGO*, porous reduced graphene oxide; *Au–Pd NF*, Au–Pd nanoflower; *CoFe*_*2*_*Se*_*4*_*/PCF*, cobalt-iron selenides embedded in porous carbon nanofibers; *MgO-MPCPE*, magnesium oxide modified pre-treated carbon paste electrode; *MWCNTPE*, multi-walled carbon nanotube paste electrode; *Ni/N-GO*, Ni/N-doped graphene oxide

### Real sample

Simultaneous determination of Hyd, Cat, and Res in tap water and river water was performed to determine the analytical applicability of the presented electrochemical sensor. Tap water samples were taken directly from the Karabük City public water system. The river water was collected from a stream (Araç Çayı) in Karabük. Water samples were spiked with standard solutions to contain two different concentrations of each analyte. The standard addition method was applied to determine Hyd, Cat, and Res concentrations in spiked samples. Recovery values were calculated and presented in Table 2. Recovery values were found to be between 93.9 and 105.5%. The results showed that the developed electrochemical sensor can be successfully utilized for the simultaneous determination of Hyd, Cat, and Res in water samples.

**Table 2:** The determination of Hyd, Cat, and Res in water samples with the c-MWCNT − Co_3_O_4_NP/PolyVal/GCE (*N* = 3)

		Added, µM	Found, µM	Recovery, %
Tap water	Hyd	50	51.8 ± 2.1	103.6 ± 4.3
		90	86.5 ± 4.2	96.2 ± 4.6
	Cat	50	49.3 ± 3.7	98.6 ± 7.4
		90	88.4 ± 1.4	98.2 ± 1.6
	Res	110	108.3 ± 6.3	98.4 ± 5.7
		190	188.1 ± 5.5	99.0 ± 2.9
River water	Hyd	50	48.8 ± 1.4	97.6 ± 2.8
		90	84.5 ± 1.2	93.9 ± 1.3
	Cat	50	50.3 ± 3.4	100.6 ± 6.7
		90	85.3 ± 4.2	94.8 ± 4.6
	Res	110	116.1 ± 4.9	105.5 ± 4.5
		190	196.8 ± 17.2	103.6 ± 9.1

## Conclusions

In this study, a nanocomposite based on c-MWCNT, Co_3_O_4_NP, and PolyVal on the GCE surface was used to construct a novel electrochemical sensor for the simultaneous determination of the dihydroxybenzene isomers Hyd, Cat, and Res. The amounts of electrode modification materials were optimized to achieve the best analytical performance from the electrochemical sensor. In addition, the pH of the working medium was optimized to determine the pH at which the best separation of the peaks of the analytes and the highest response was obtained. Individual and simultaneous determinations of Hyd, Cat, and Res were performed under optimum conditions. The wide linear operating range, good sensitivity, reproducibility, and selectivity in the simultaneous determination of dihydroxybenzene isomers with c-MWCNT − Co_3_O_4_NP/PolyVal/GCE were attributed to the synergistic effect of the modification materials. c-MWCNT provides a high surface area and excellent electrical conductivity, facilitating rapid electron transfer. Co_3_O_4_NP contributes remarkable electrocatalytic activity, promoting efficient redox reactions. PolyVal serves as a polymeric matrix that not only offers functional groups that can interact with the analyte but also supports stable immobilization of the nanomaterials on the electrode surface. The combination of these materials results in a modified surface with improved conductivity, a larger electroactive area, and enhanced catalytic activity, leading to improved sensor performance. Furthermore, satisfactory recovery values were obtained for the simultaneous determination of Hyd, Cat, and Res in tap water and river water with the electrochemical sensor. The results obtained suggest that c-MWCNT − Co_3_O_4_NP/PolyVal/GCE will contribute to the literature as a reliable, efficient, sensitive, repeatable, selective, and simple method for the simultaneous determination of Hyd, Cat, and Res.

## Data Availability

Data will be made available on request.
